# The Antimicrobial Efficacy of Copper Complexes: A Review

**DOI:** 10.3390/antibiotics14050516

**Published:** 2025-05-16

**Authors:** Kwanele Ngece, Vuyolwethu Khwaza, Athandwa M. Paca, Blessing A. Aderibigbe

**Affiliations:** Department of Chemical and Earth Sciences, University of Fort Hare, Alice 5700, Eastern Cape, South Africa; 201102901@ufh.ac.za (K.N.); blessingaderibigbe@gmail.com (B.A.A.)

**Keywords:** copper complexes, antimicrobial activity, antibacterial, antifungal, bioinorganic chemistry, mechanism of action, metal-based drugs

## Abstract

The alarming increase in antimicrobial resistance has intensified the search for novel therapeutic agents capable of combating resistant microbial strains. Copper complexes have emerged as promising antimicrobial agents due to their intrinsic redox activity, ability to disrupt microbial membranes, and interactions with vital biomolecules such as DNA and proteins. This review critically evaluates the antimicrobial potential of copper complexes reported between 2018 and 2025, emphasizing their structural diversity, mechanisms of action, and biological performance against a wide range of bacterial and fungal pathogens. Key findings reveal that Schiff base copper complexes, amino acid derivatives, heterocyclic ligands, and mixed-ligand systems exhibit potent antimicrobial activities, often surpassing standard antibiotics. Mechanistically, copper complexes induce reactive oxygen species (ROS) generation, inhibit enzyme function, cause DNA cleavage, and compromise cell membrane integrity. Furthermore, structure–activity relationship (SAR) analyses indicate that ligand type, coordination geometry, and lipophilicity significantly influence antimicrobial efficacy. Overall, the reviewed studies support the development of copper-based compounds as viable candidates for antimicrobial drug development. This review also identifies current challenges and gaps in knowledge, such as limited in vivo studies and toxicity assessments, which must be addressed to advance these compounds toward clinical application.

## 1. Introduction

**Figure 1:**
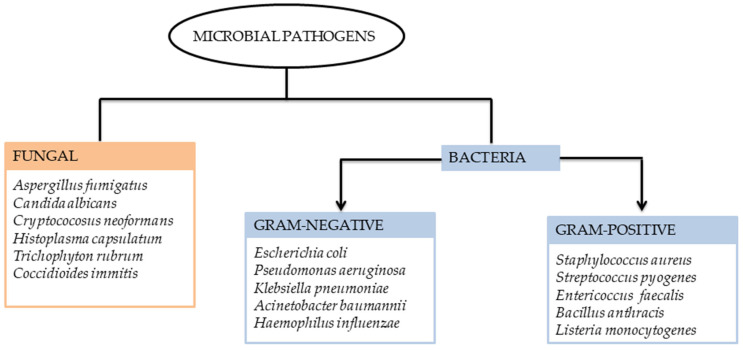
Classification of common microbial pathogens.

Microbial infections are mostly caused by microbes that develop resistance to previously administered treatments [1]. Super-infections that occur after primary infections can significantly complicate microbial diseases and may lead to severe outcomes [2]. Intensive care unit (ICU) provides a setting for persistent microorganisms that are difficult to treat. Patients in the ICU have immunosuppression, comorbidities, and seniority, and often undergo invasive procedures [3]. Infections such as respiratory viral infections frequently result from co- and super-infections. Research from clinical, laboratory, and epidemiological studies suggests that when patients with viral infections have co-infections with other viruses, it can significantly raise their risk of high mortality [4]. Evidence shows that bacterial and fungal co-infections can substantially increase the severity of viral infections, leading to prolonged hospitalization, increased need for intensive care, and higher mortality rates, thereby worsening overall patient prognosis [5]. Additionally, intracellular pathogens can also lead to these co-infections. On the other hand, super-infections include hospital-acquired pulmonary infections that develop after the patient has been admitted for more than 48 h [6,7]. Excessive antibiotic use is associated with the emergence and spread of multidrug-resistant (MDR) infections [8]. One or more community-acquired co-infections have been reported to influence 3–7% of COVID-19 cases [4]. Approximately 2.5–5% of these co-infections were associated with bacterial infections, namely *S. epidermidis*, *methicillin-sensitive S. aureus*, *E. faecalis*, *E. coli*, *methicillin-resistant S. aureus* (MRSA), *Proteus mirabilis*, and *K. pneumoniae* [9,10]. The two most common strains of bacteria that cause co-infections are *S. pneumoniae* and *S. aureus* [7]. Opportunistic invasive fungal infections are frequently identified as community-acquired co-infections in patients with COVID-19 [11]. Aspergillosis and candidiasis (*C. albicans*, *C. glabrata*) contribute to the most frequent fungal co-infections, but *mycormycosis*, *cryptococcosis*, and other fungal pathogens have also been detected [12]. Antimicrobial resistance (AMR) is projected to emerge as the foremost cause of mortality worldwide in the upcoming decades. In 2019, it was estimated that AMR was linked to approximately 4.95 million deaths, with 1.3 million of those deaths being directly due to resistant infections [13]. This projection indicates that annual deaths could rise to 10 million in the next few years [13]. This surge is partly attributed to the extensive overprescription of antibiotics for COVID-19 patients over the last two years [13]. Among the various approaches explored, metal-based compounds, particularly those involving copper, have gained considerable attention for their potent antimicrobial activity and unique mechanisms of action. Microbial infections caused by various pathogens, including Gram-positive and Gram-negative bacteria and fungi, remain a major global health threat (Figure 1). These pathogens are responsible for multiple infections and have shown increasing resistance to conventional therapies, necessitating the exploration of alternative agents such as metal-based complexes.

Copper, an essential trace element involved in various biological processes, exhibits redox properties that enable it to interact with biomolecules in microbial cells, leading to oxidative stress, membrane damage, and DNA degradation [14,15]. When complexed with organic ligands, copper’s antimicrobial efficacy can be significantly enhanced through increased stability, selective targeting, and improved cellular uptake [16]. Numerous copper complexes, including those formed with Schiff bases, amino acids, heterocycles, and natural products, have shown promising antimicrobial properties.

This review focuses on the antimicrobial efficacy of copper complexes, with particular emphasis on their structural diversity, modes of action, and potential biomedical applications. The review highlights studies published from 2018 to 2025, encompassing mononuclear and polynuclear copper complexes derived from various ligands, including Schiff bases, azoles, and other biologically relevant scaffolds. Particular attention is given to their activity against bacterial and fungal pathogens, the underlying mechanisms of antimicrobial action, and structure–activity relationships (SAR). The aim is to provide an updated and critical overview of the advancements in this field, identify key trends and knowledge gaps, and suggest directions for future research in the development of copper-based antimicrobial agents.

## 2. Methodology

To ensure a comprehensive and systematic literature review, relevant peer-reviewed articles on the antimicrobial properties of copper complexes published between 2018 and 2025 were identified. Electronic databases, including PubMed, Scopus, Web of Science, and Google Scholar, were used for literature retrieval. The following keywords were applied in various combinations: “copper complexes”, “antimicrobial activity”, “antibacterial”, “antifungal”, “bioinorganic chemistry”, “mechanism of action”, and “metal-based drugs”.

Inclusion criteria were as follows: (i) studies published in English; (ii) articles that reported the synthesis, characterization, and antimicrobial evaluation of copper complexes; (iii) reviews and original research articles presenting relevant biological and mechanistic insights. Exclusion criteria included non-English papers, patents, conference abstracts, and studies lacking antimicrobial data.

The selected published studies were analyzed to highlight current trends, structural diversity, biological performance, and proposed mechanisms of action of copper-based compounds against microbial pathogens.

## 3. Copper

**Figure 2:**
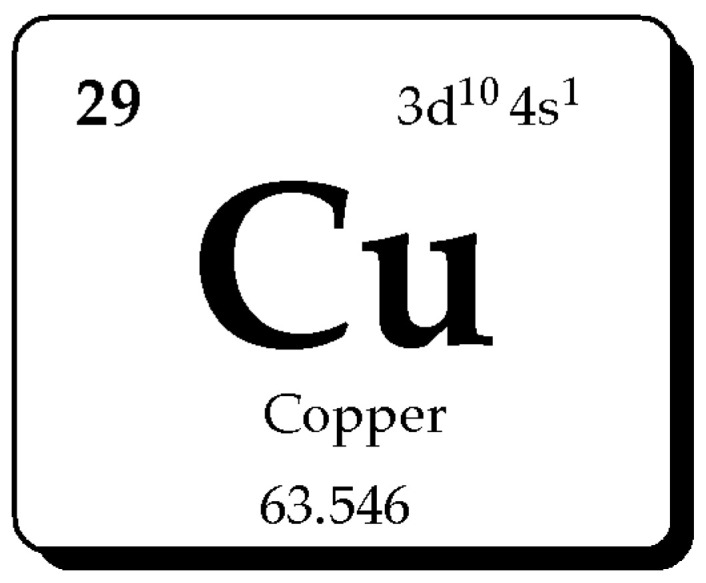
Copper on the periodic table.

Copper (Cu) (Figure 2) is an essential trace valuable element for living organisms, playing crucial roles in various metabolic pathways, such as antioxidant activity, maintaining tissue integrity, iron metabolism, supporting synaptic function, and respiratory functions [17]. Copper is an essential biometal involved in various physiological processes, including redox regulation, enzyme function, and antimicrobial defense, making it a promising candidate for developing metal-based therapeutics [18]. Research shows that copper (Cu) is unevenly distributed across the Earth [19]. Its concentration is about 68 parts per million (ppm) in the Earth’s crust and 0.003 ppm in seawater. Approximately 1.0 ppm of copper is present in the human body [20]. In biological systems, copper can exist in two oxidation states: Cu^+^ and Cu^2+^. It has a low redox potential of 0.158 V between these states in water, allowing it to exchange electrons with other systems [19]. In fluids, copper primarily exists in its Cu(II) state, whereas within the intracellular reducing environment, it is predominantly found in its Cu(I) state [21]. Copper is the sole hard surface material that has gained approval from the U.S. Environmental Protection Agency (EPA) due to its antimicrobial properties [22]. In 2008, the EPA formally acknowledged copper and its alloys as the pioneering metallic antimicrobial agents [23]. They certified copper’s strength to eradicate 99.9% of pathogenic bacteria within 2 h. Since then, significant advancements have been made in understanding copper’s bactericidal properties, termed ‘contact killing’, which enables the swift elimination of harmful bacteria [24]. Copper is essential for numerous biological processes, functioning primarily as a catalytic cofactor in metalloenzymes such as cytochrome C oxidase (involved in cellular respiration), superoxide dismutase (antioxidant defense), ceruloplasmin (iron metabolism), and lysyl oxidase (collagen cross-linking in connective tissues). In the immune system, copper regulates T-cell proliferation, neutrophil function, and cytokine production, while in the nervous system, it supports neurotransmitter synthesis and myelination. Copper also plays a critical role in bone health by aiding in collagen formation and in hematopoiesis by facilitating iron mobilization and erythropoiesis [25,26,27,28,29]. It plays vital roles not only in various synthetic pathways, but it is also essential to note that irregularities in its levels can lead to certain diseases. For instance, Menkes and Wilson’s syndrome results from deficiency (due to a mutation in the copper-transporter gene ATP7B on the chromosome) or an excess concentration of copper (caused by a mutation in the ATP7A gene on the chromosome), respectively [20].

The human body typically contains around 100 mg of copper on average [30]. Excess free Cu within the cell poses a significant risk of damaging nucleic acids, membranes, and proteins. To prevent undesirable reactions and maintain homeostasis, copper is conjugated to chaperone proteins [31]. The high-affinity transmembrane transporter, Copper Transporter 1 (CTR1), facilitates the entry of copper (Cu) into cells. Beforehand, it undergoes reduction to its Cu(I) state by the enzyme STEAP metalloreductase (human 6-transmembrane epithelial antigen of prostate reductase) [32]. Upon entering the cells, copper latches onto various chaperones, specifically responsible for its transport to copper proteins. For instance, cytosolic CCS (copper chaperone for SOD1) effectively delivers copper to SOD1 (Cu, Zn-superoxide dismutase). Copper can become cytotoxic if its levels exceed cellular requirements or if it is improperly distributed within the cell [33]. Consequently, both cells and organisms have highly regulated mechanisms to manage the delivery and allocation of copper under normal conditions [34].

### 3.1. Copper Complexes

**Figure 3:**
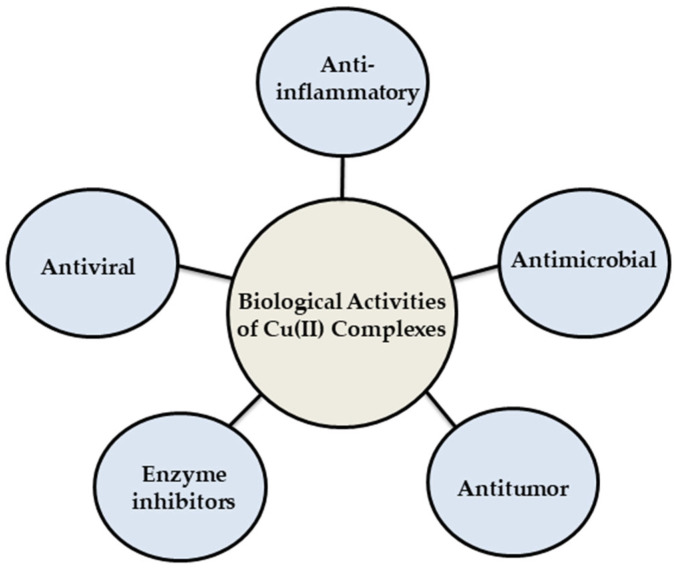
Some biological activities of Cu(II)complexes.

In recent times, there has been growing interest in developing metal-based drugs with pharmacological potential. The thermodynamic and kinetic properties of these complexes play a crucial role in determining the biological activity of bio-metals [35]. Additionally, developing chelates in vivo can enhance the lipophilicity of drugs, significantly improving their ability to penetrate the target site, thereby increasing their effectiveness [36]. Copper complexes have garnered significant attention in coordination chemistry, technical applications, and catalysis. This interest stems from their unique spectroscopic properties, anion selectivity, and notable biological significance [36]. Copper(II) complexes represent a significant class in chemistry due to their intriguing coordination chemistry, which includes flexible redox properties, diverse geometries, and various oxidation states [37,38]. They have extensive applications across multiple fields, such as catalysts in oxidation [39], reduction, and epoxidation reactions [40]. Moreover, copper(II)-based compounds display a wide range of biological activities (as shown in Figure 3) and have been extensively investigated in the medical field for their potential in treating various diseases [41], which are intimately connected to their capacity to form metal complexes. For instance, when coordination occurs, the lipophilicity, which influences the rate of cellular entry, is altered. This phenomenon can be elucidated through forming coordination bonds, as Overtone’s concept and Tweedy’s chelation theory. During coordination formed by donor groups, the lipophilicity of the complexes is improved, which depends on the removal of π-electrons over the chelate ring system. This increased lipophilicity improves their ability to permeate the lipid layer of microorganisms, thereby leading to more efficient destruction [42,43]. This modification can reduce specific side effects and a novel range of bioactive properties not displayed by the free ligand [44].

**Figure 4:**
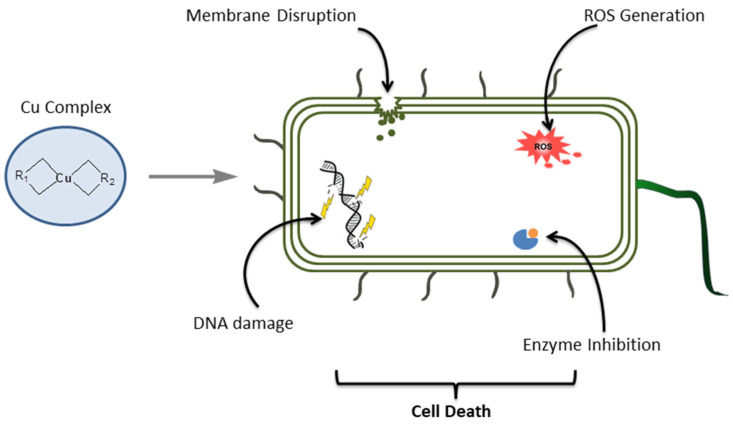
Cu(II) complex mode of action against microbial cells.

According to Zaki et al. [45], metal-based compounds can be arranged into seven categories depending on the function of the metal and ligand moieties: (1) the metal complex is active in inert form, (2) the metal complex is active in its reactive form, (3) the metal serves as a radiation enhancer, (4) the compound contains a radioactive metal, (5) the metal or its biotransformation product is active, (6) a ligand is biologically active, and (7) only a fragment of the complex is active [45]. Three-dimensional configurations of metal complexes formed by the coordination of organic ligands with metal allow the complexes to react more effectively with biological molecules. Therefore, in addition to the metal, the type of ligand has a significant effect on the chemical activities and subsequent medical applications of metal complexes, so the change of ligand can lead to changes in the photophysical, electrochemical, spectroscopic, and biological properties of the complexes [46]. There are different mechanisms of action that depend on the geometry of the complexes and the nature of the ligand [47]. They can act against microorganisms by occupying surface sites, which would typically be utilized in initiating the infection of the host cell, preventing the first step in the infection [48]. Alternatively, other mechanisms involve the effects on the cell wall or membrane, interaction with DNA [49], binding or inhibition of enzymes and membrane proteins [50], and the generation of reactive oxygen species (ROS) (see Figure 4) [13]. This review delves into the antimicrobial capabilities of copper complexes. By compiling recent progress in copper complex research, we aim to shed light on the effectiveness and potential of supplementary treatments for tackling microbial infections.

#### 3.1.1. Antibacterial Activity of Cu(II) Complexes

**Figure 5:**
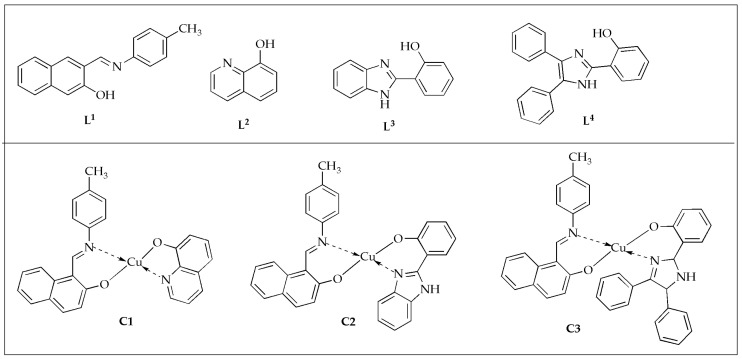
Chemical structures of Cu(II) complexes (**C1**–**C3**) and their corresponding ligands (**L^1^**–**L^4^**).

The antimicrobial activity of heteroleptic Cu(II) complexes with the general formula [Cu(**L^1^**)(**L^n^**)] where **L^1^** is 1-{[(4-methylphenyl)imino]methyl}-2-naphthol as the primary ligand, and **L^n^** represents secondary ligands **L^2^** = 8-hydroxyquinoline, **L^3^** = 2-(1H-benzimidazol-2-yl)phenol, and **L^4^** = 2-(4,5-diphenyl-1H-imidazol-2-yl)phenol (Figure 5) was evaluated by Ismael et al. against selected Gram-negative and Gram-positive bacteria, as well as fungal strains. [51]. In their study, they found that the complexes outperformed the bare equivalent ligands in terms of their antimicrobial activity against specific bacteria and fungi by around 3.37 (2.99) times for complex **C1** (Cu**L^2^**) and more significant than the bare **L^1^** ligand (**L^2^** co-ligand) against *A. fumigatus* at 100 µg/mL (Table 1). The inhibition zone diameters increased in the following order: **L^1^** < **L^4^** < **L^2^** < **L^3^** < **C3** < **C2** < **C1**. The activity was further assessed using minimum inhibitory concentrations (MIC) measurements. In these evaluations, the synthesized complexes demonstrated enhanced potency with lower MIC values than the free ligands. Complex **C2** demonstrated the lowest MIC with values ranging between 20.50 and 22.50 ppm against *E. coli*, *B. cereus*, and *A. fumigatus*. The condensation of p-toluidine with 2-hydroxy-naphthaldehyde yielded the primary ligand (**L^1^**). Incorporating 8-hydroxy-quinoline (**L^2^**) significantly enhanced the antimicrobial activity of the Cu(II) mixed-ligand complex (Cu**L^2^**) [51].

**Figure 6:**
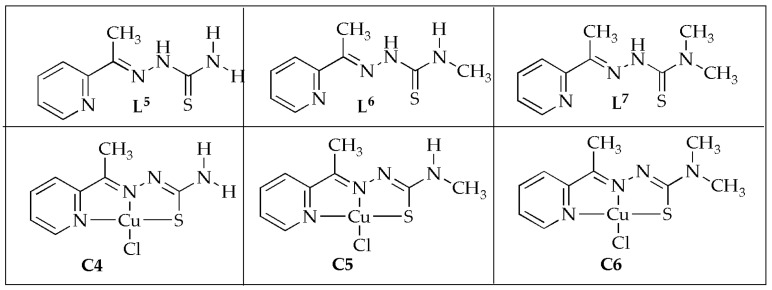
Chemical structures of Cu(II) complexes (**C4**–**C6**) and their corresponding ligands (**L^5^**–**L^7^**).

The evaluation of the copper(II) thiosemicarbazone complexes (**C4**–**C6**) (Figure 6) against selected G^+^/G^−^ bacterial strains by Qi et al. [52] displayed promising findings. These Cu(II) complexes demonstrated greater antimicrobial effectiveness against G^−^ bacteria comparable to G^+^ bacteria. The complexity of the cell wall structure played a significant role in influencing the bacterial potency of the complexes. Compound **C4** was the most potent antibacterial agent against all tested G^+^/G^−^ bacterial strains, and the enhanced antibacterial effect was attributed to the presence of two methyl groups substituted on the nitrogen, which resulted in a tertiary amine. Additionally, against *S. aureus,* the ligands displayed no inhibition zones and exhibited inhibition zone diameters of 12.22 mm (**L^6^**) and 13.74 mm (**L^7^**) against *E. coli*, respectively (Table 2). The complexes showed improved inhibitions against *S. aureus* and *E. coli* with diameters between 14 and 25 mm. Notably, the complexes displayed excellent antibacterial properties against *E. coli*. These findings are advantageous in advancing the development of novel Cu(II) complexes as potential antitumor drug candidates [52].

**Figure 7:**
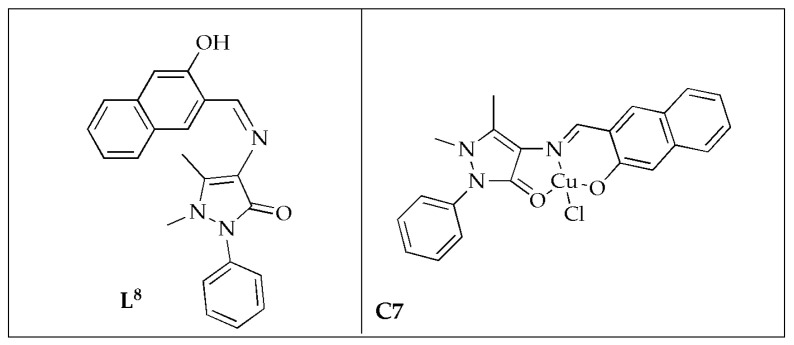
Chemical structures of ligand (**L^8^**) and its pyrazole nucleating Cu(II) complex **C7**.

The antimicrobial efficacy of pyrazole nucleating copper(II) (Figure 7) complex **C7** was evaluated by Azam et al. [53]. Antimicrobial analysis indicated that the pyrazole nucleating ligand (**L^8^**) was less effective than its synthesized complex when tested against various pathogenic microorganisms. The studied compounds exhibit varying inhibition zones when tested against different microbial strains. Additionally, the results indicated that complex **C7** exhibited superior antibacterial activity against *S. sonnei*, *B. subtilis*, and *P. aeruginosa* microbes, with an inhibition zone of 21 mm and an MIC value of 62.5 µg/mL (Table 3). This performance surpassed that of the standard drug, Amoxicillin. Incorporating the metal ion into the pyrazole nucleating Schiff base ligand (**L^8^**) significantly improved its antibacterial effectiveness. As a result, the new pyrazole nucleating Cu(II) complex **C7** emerges as a promising candidate for antimicrobial drugs [53].

**Figure 8:**
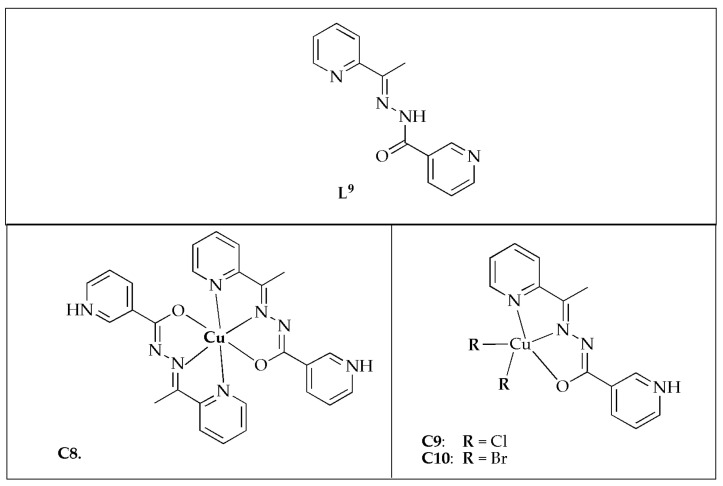
Chemical structures of Cu(II) complexes (**C8**-**C10**) and their corresponding ligand 2-cetylpyridinenicotinichydrazone (**L^9^**).

SanSantiago et al. [54] synthesized copper(II) complexes with 2-cetylpyridinenicotinichydrazone (**L^9^**) (Figure 8) and assessed their antibacterial activity against various selected bacterial strains. The antibacterial effectiveness of free ligand **L^9^** demonstrated significant potency against *S. mutans* and *S. salivarius*, with MIC values of 1.56 µg/mL and 312 µg/mL, respectively. The Cu(II) complexes exhibited limited antibacterial potency against *E. faecalis*, except for complexes **C9** and **C10**, with MIC values of 100 µg/mL. However, they demonstrated moderate to good activity against other bacterial strains, with complex **C10** showing the best results, achieving a MIC value of 3.12 µg/mL against *S. mutans* (Table 4). Additionally, the hydrazone and its Cu(II) complexes exhibited superior MIC values comparable to the positive control. Generally, upon coordination with Cu(II) ion, the antibacterial potency of **L^9^** was enhanced. However, against *S. mutans* and *S. salivarius*, the hydrazone demonstrated comparable or superior effectiveness than the complexes. The observed enhancement might be attributed to the increased lipophilicity resulting from the sharing of the Cu^2+^ charge with donor groups upon coordination and its reduced polarity. This enhances the complexes’ interchangeability with proteins comparable to free ligands. Additionally, the construction of ROS within the intracellular environment could contribute to the enhanced activity. The antibacterial effectiveness of complexes **C9** and **C10** surpassed that of complex **C8** against most of the investigated pathogens. The ESI-MS analysis disclosed that the emergence of cationic species [CuX(**L^8^**)]^+^ in solution might play a role in the antibacterial activity of complexes **C9** and **C10**. The study concluded that the assessed compounds exhibited antibacterial efficacy, with the coordination of the Cu(II) atom significantly enhancing the potential of **L^8^** against most of the tested bacterial strains [54].

**Figure 9:**
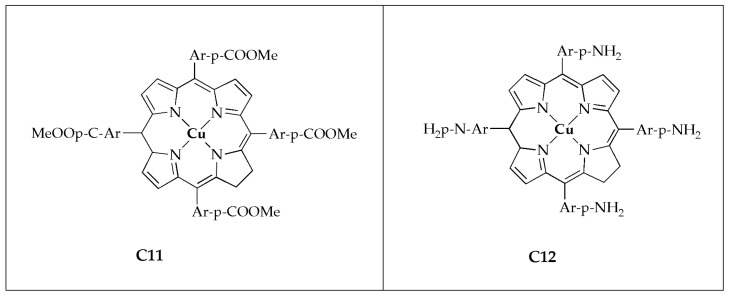
Chemical structures of the Cu(II) complexes (**C11** and **C12**).

Beyene and Getaneh [55] reported the antibacterial effectiveness of metalloporphyrins; 5, 20, 15, 20-tetrakis (para-X phenyl)porphyrinato Cu(II) (Figure 9) and assessed their activity against selected G^−^/G^+^ bacteria. The studied complexes exhibited improved antibacterial growth inhibition potency comparable to the porphyrin ligand. The incorporation of the metal ion clearly suggests it is the ideal candidate for the inhibition of bacterial growth (Table 5). The significant antibacterial effectiveness of transition metal complexes of porphyrins is attributed to two main factors: The Overtone concept and Tweedy’s chelation theory [42,43]. As the concentration of the complexes rises, their antimicrobial efficacy likewise enhances. Metal complex **C11** with the electron-withdrawing group (-COOMe) demonstrated superior activity than complex **C12** with the electron-donating group (-NH_2_). Additionally, it was observed that metalloporphyrins exhibit greater bacterial growth inhibition comparable to metal salt or DMSO. It has been demonstrated that metalloporphyrins with an electron-withdrawing group at the para-positions exhibit superior antibacterial activity compared to those with an electron-donating group at the same positions. These findings suggest that metalloporphyrin derivatives hold significant promise as effective antibacterial agents [55].

**Figure 10:**
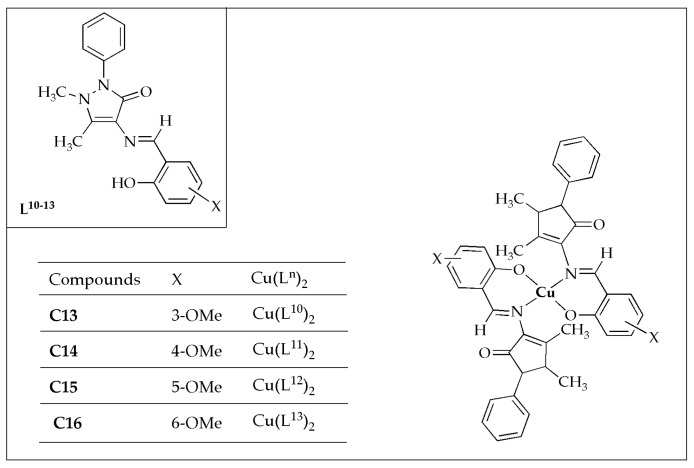
Chemical structures of Cu(II) complexes (**C13**–**C16**) and their Schiff base ligands (**L^10^**–**L^13^**).

The biological potency of a novel series of Cu(II) complexes (**C13**–**C16**) with four Schiff-based ligands (Figure 10), synthesized by Kargar et al. [56], was evaluated against selected bacterial microbes. All the complexes investigated exhibited considerable antibacterial efficacy against *S. aureus* and *E. coli*, whereas the free ligands demonstrated only moderate efficacy (Table 6). All assessed complexes exhibited superior antibacterial potency against G^+^ strains compared to G^−^ strains. This difference is attributed to the thicker peptidoglycan layer in G^+^ bacteria, which enables them to be readily absorbed. However, G-bacteria are shielded from certain physical invasions since they do not absorb the surrounding foreign materials. Altering only the methoxy site, the synthesized Schiff-based ligands exhibited a wide range of inhibitory effects on the growth of the strains (MIC values ranging from 64 to 512 µg/mL against *E. coli* and *S. aureus*), thus indicating a significant influence of modifications on antibacterial efficacy. The complexes exhibit superior bactericidal activity and inhibitory effects compared to their ligands, within a narrow range: MBC and MIC values are 128 and 64 µg/mL against *E. coli*, and 64 and 32 µg/mL against *S. aureus*, respectively. The Cu(II) complexes demonstrate a significant antibacterial effectiveness comparable to the parent Schiff base ligands [56].

**Figure 11:**
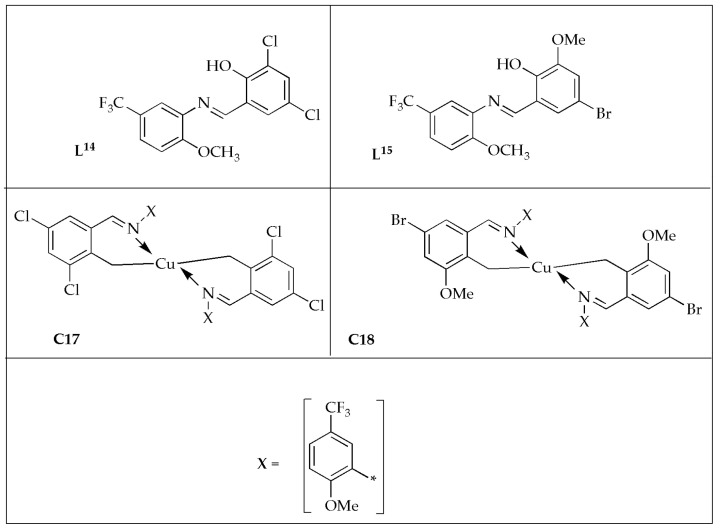
Chemical structures of Ligands (**L^14^**, **L^15^**) and Cu(II) 2-[(5-(trifluoromethyl)-2-methoxyphenylimino)methyl)]-4,6-dichlorophenol (**C17**) and Cu(II) 2-(5-(trifluoromethyl)-2-methoxyphenylimino)methyl)-4-bromo-6-methoxyphenol (**C18**).

Jyothi et al. [57] formulated novel [Cu(**L^14^**)_2_] (**C17**) [Cu(**L^15^**)_2_] (**C18**) complexes (Figure 11) and reported their antibacterial effectiveness against selected bacterial strains. The free ligands exhibited moderate effectiveness against bacterial strains, whereas the Cu(II) complexes displayed good effectiveness comparable to the controls (Table 7). The antibacterial potency was enhanced when the ligands were coordinated with the Cu(II) ion. The enhanced antibacterial effectiveness of the complexes may be attributed to the high lipophilicity of the metal complex [55,58]. Antibacterial results displayed that the complexes exhibited significantly more antibacterial potency than the corresponding Schiff bases but lower potency than the controls. Developing novel metal-based drugs is a potential path in treating bacterial infections.

The antibacterial studies demonstrate that Cu(II) complexes consistently exhibit improved activity compared to their corresponding free ligands. This enhancement is attributed to factors such as increased lipophilicity, better membrane permeability, and the ability of copper to participate in redox reactions, potentially leading to ROS generation in microbial cells. Complexes **C1**–**C3**, incorporating mixed ligands, showed superior potency compared to individual components, highlighting the role of synergistic ligand interactions. Thiosemicarbazone-based complexes (**C4**–**C6**) were particularly effective against Gram-negative bacteria, with structural features such as methyl substitution enhancing their efficacy. Pyrazole-based complex **C7** and hydrazone-derived complexes **C8**–**C10** further emphasized that copper coordination improves antimicrobial action, likely due to increased bioavailability and interaction with bacterial biomolecules. Schiff base and porphyrin complexes (**C11**–**C16**) demonstrated that the electronic effects of substituents (e.g., -COOMe vs. -NH_2_) significantly impact antibacterial outcomes.

Overall, structural variations among ligands, combined with copper’s coordination properties, contribute to the observed antimicrobial trends. These findings support the development of Cu(II) complexes as promising antibacterial agents, though further in vivo and mechanistic investigations are needed to validate their therapeutic potential.

#### 3.1.2. Antifungal Activity of Cu(II) Complexes

**Figure 12:**
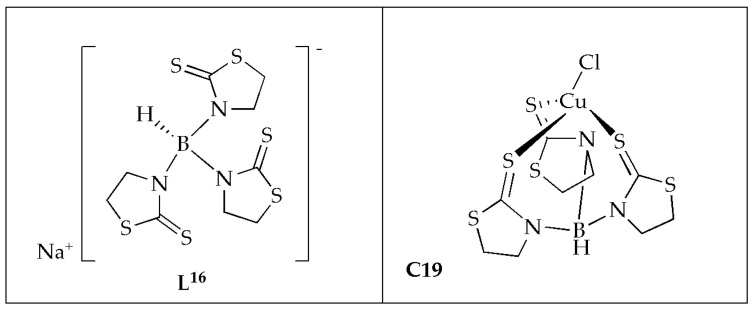
Structure of Cu(II) complex **C19** and its corresponding hydrotris(2-mercaptothiazolyl)borate ligand (**L^16^**).

Ferreira et al. [59] synthesized Cu(II) complex coordinated with scorpionate ligand in a tridentate mode (Figure 12) and evaluated the antifungal activity against microorganisms of clinical importance. The MIC values ranging from 66 to 260 µmol/L^−1^ demonstrate that complex **C19** exhibited the highest antifungal effectiveness. Complex **C19** demonstrated fungicidal potency against *C. albicans* and *C. glabrata*, with an MFC of 260 µmol/L^−1^ (Table 8). The Cu(II) complex exhibited superior antifungal activity, four times more effective than ketoconazole against *C. albicans*. The inclusion of the transition metal enhanced the therapeutic index of antifungal substances in vitro. When assessing cytotoxicity in Vero cells, it showed a reduced potential for cellular injury, indicating a low risk of nephrotoxicity. The selectivity of Cu(II) complex ranged between 8 and 16 times more for *C. krusei* and *C. albicans,* comparable to Vero cells. These findings suggest that copper complexes are linked to enhanced antifungal activity and diminished toxicity. Given the challenge of synthesizing drugs that selectively target fungal strains due to their similarity to mammalian cells, Cu(II) coordination strategies appear favorable [59].

**Figure 13:**
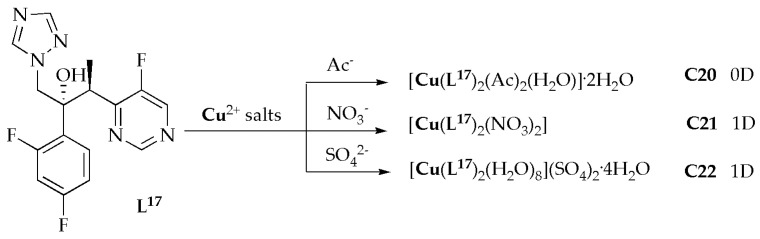
Cu(II)-based metal coordination of compounds (**C20**–**C22**).

Copper(II)-based metal coordination complexes **C20**–**C22** (Figure 13) with voriconazole ligands (**L^17^**) were synthesized and evaluated for their antimicrobial potency against selected fungal strains by Zhao et al. [60]. The antifungal results for complexes **C20**–**C22** demonstrated varying levels of efficacy in inhibiting different types of *Candida*. Notably, they showed potent inhibitory activity against *C. glabrata* and *C. neoformans*. Amongst the complexes, **C20** notably displayed the highest effectiveness against *Candida* spp., with exceptional MIC values ranging from 0.05 to 0.2 µg/mL (Table 9). Furthermore, the free ligand **L^17^** had MIC values higher than most complexes, suggesting that the complexes exhibit greater efficacy against *Candida* spp. comparable to the free ligand voriconazole. The antifungal assessments showed that complexes **C20** through **C22** are highly effective against a range of *Aspergillus* spp. Notably, complex **C20** demonstrated the most potent inhibition of fungal effectiveness, with MIC values of 0.05, 0.05, 0.1, and 0.2 µg/mL for *A. niger*, *A. flavus*, *A. fumigatus*, and *A. terreus*, respectively. These values are either superior to or comparable to those of voriconazole. Significantly, the antifungal activities of complexes **C20**–**C22** are either more potent or comparable to the free ligand. As previously mentioned, complex **C20**, which contains CH_3_COO^−^, exhibited mononuclear motifs, whereas complex **C21,** with a NO_3_^−^ ligand, and complex **C22,** with a SO_4_^2−^, presented polymeric structures. Complex **C20** showed higher inhibitory efficiency than **C21** and **C22** complexes. Notably, three new Cu(II)-based metal complexes feature 0D and 1D motifs. They demonstrated exceptional inhibitory efficiency against the fungal species, indicating their potential applications in the antimicrobial field. The findings of this study serve as an outstanding example of constructing metal coordination complexes with biological activities [60].

**Figure 14:**
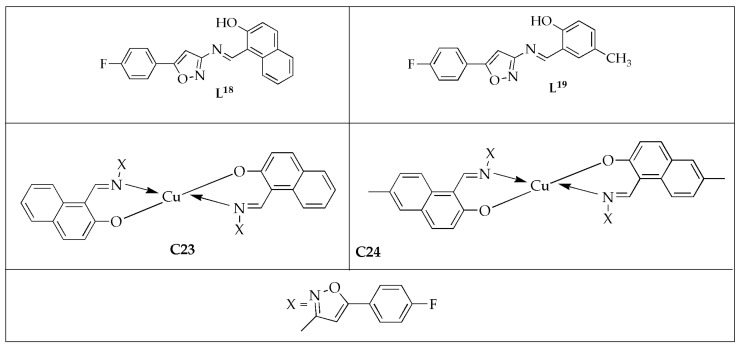
Chemical structures of bivalent Cu(II) complexes (**C23**, **C24**) and their corresponding ligands (**L^18^**, **L^19^**).

Ramesh et al. [61] assessed the antifungal effectiveness of bivalent metal complexes [Cu(**L^17^**)_2_] (**C23**) and [Cu(**L^18^**)_2_] (**C24**) with newly synthesized Schiff bases **L^18^** = (1-(5-(4-flourophenyl)isoxazole-3-ylimino)methyl)napthalen-2-ol), **L^19^** = (2-(5-(4-flourophenyl)isoxazole-3-ylimino)methyl)-4-methylphenol) (Figure 14), against selected fungal strains [61]. Mancozeb was explored as a standard drug for antifungal activity. It was reported that Schiff base ligands displayed moderate effectiveness against fungal strains, while their corresponding Cu(II) complexes exhibited higher activity than the free Schiff base ligands. This enhanced activity of the complexes can be attributed to the chelate effect. Based on the inhibition zone diameter, complex **C23** demonstrated the highest activity against *Macrophomina phaseolina* (*M. phaseolina*) and *Sclerotium rolfsii* (*S. rolfsii*) when compared to complex **C24**. However, it was less effective than the standard drug. Naphthalen-2-ol in complex **C23** contributed to its greater antifungal activity than **C24**, which contained 4-methylphenol. The tabulated results indicate that Cu(II) complexes exhibited superior potential effectiveness against the specified fungal species (Table 10). This is likely due to the atomic radius and electro-negativity of the Cu(II) ion. Additionally, factors such as dipole moment, solubility, complex geometry, stereochemistry, coordination sites, concentration, and hydrophobicity also contribute to the antifungal potency of these complexes. The antifungal activity of the Schiff base ligand and its metal complexes highlights that metal complexes have greater antifungal potency than the Schiff bases alone [61].

Azam et al. [53] prepared complex **C7**. Figure 7 containing a pyrazole nucleating ligand (**L^8^**) with Cu(II) ion, and investigated the antifungal activity against selected fungal strains. The analysis demonstrated that the pyrazole nucleating ligand, **L^8^**, exhibited lower activity against the tested pathogenic microorganisms compared to its synthesized complex **C7**. Complex **C7** demonstrated superior efficacy, with inhibition zones ranging from 17 to 25 mm (MIC = 125 µg/mL), while the free ligand L^8^ had inhibition zones of 8–13 mm (MIC = 500 µg/mL) (Table 11). Additionally, complex **C7** exhibited good activity when compared to fluconazole, with inhibition zones of 20–28 mm (MIC = 125 µg/mL) against all the tested fungal pathogens. The complex effectively inhibited the growth of fungal strains *P. notatum*, *A. niger*, and *A. flavus*, resulting in an expansion of the inhibition zone to approximately 23 mm (125 µg/mL). The Cu(II) complex exhibited superior antifungal potency against *A. flavus* when compared to Fluconazole (control). The coordination of the ligand with the Cu(II) ion significantly improved their activity against fungal strains, showing the potential of copper complexes for antifungal applications [53].

**Figure 15:**
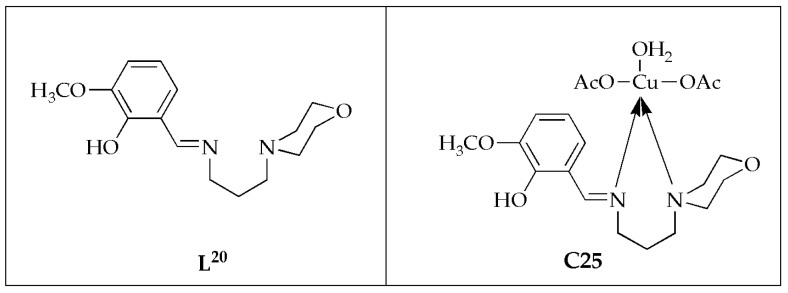
Chemical structure of bidentate-morphine-based ligand (**L^20^)** and its Cu(II) complex **C25**.

The antifungal effectiveness of water-soluble Cu(II) complex **C25**, from bidentate-morpholine based ligand (L-morpholinopropylimino)methyl)-6-methoxyphenol), was synthesized and investigated against *C. albicans* and *A. niger*, by Senthilkumar et al. (Figure 15) [62]. The study demonstrated that the Cu(II) complex **C25** displayed greater antifungal activity than the ligand **L^20^**. This enhanced activity was attributed to factors such as the size and charge distribution of the metal ion, as well as the shape and redox potential of the metal chelates. Based on the zone of inhibition values, it was reported that complex **C25** (23.7 ± 1.19 mm against *C. albicans* and 19.8 ± 0.99 mm against *A. niger*) was more potent than the ligand (13.3 ± 0.67 mm against *C. albicans* and 11.7 ± 0.59 mm against *A. niger*) (Table 12). However, it was less active when compared to amphotericin (control), which had inhibition zones of 28.7 ± 1.44 mm against *C. albicans* and 28.6 ± 1.43 mm against *A. niger*. The observed inhibition zones for these species follow this order: **L^20^** < **C25** < amphotericin. As confirmed by the results, groove binding is the expected route for Cu(II) complex to interact with DNA. Molecular docking results indicated that the prepared ligand and the Cu(II) ion can interact with the DNA helix. This study confirmed that the synthesized Cu(II) complex **C25** exhibits enhanced antifungal efficacy compared to the free ligand [62].

**Figure 16:**
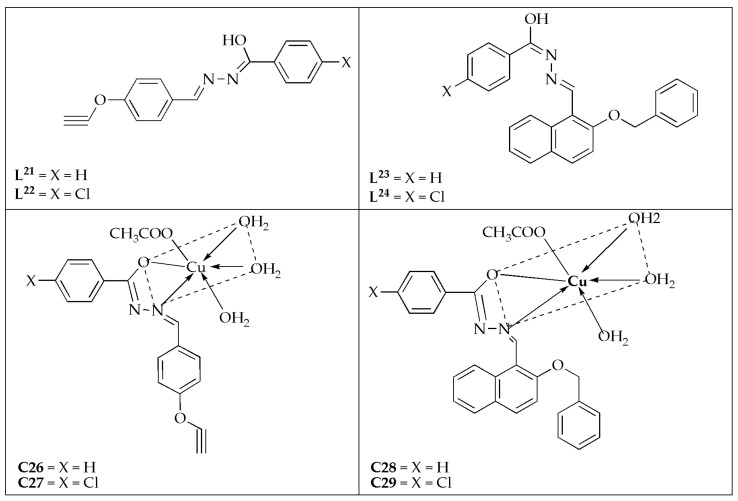
Chemical structures of and their Cu(II) complexes **C26**–**C29** and their corresponding Schiff base ligands (**L^21^**–**L^24^**).

Yadav et al. [63] synthesized a series of Cu(II) complexes prepared by four novel Schiff base ligands (**L^21^**–**L^24^**) (Figure 16) and investigated their antifungal effectiveness against *C. albicans* and *A. niger*. The antifungal efficacy displayed by the Schiff bases follows the order: **L^21^** < **L^22^** < **L^23^** < **L^24^** against the fungal strains. This activity pattern is likely related to their molecular weights, which follow the same sequence. The synthesized complexes were more effective on *C. albicans* with MIC values between 0.0052 and 0.0068 µM/mL compared to *A. niger* (MIC values between 0.0102 and 0.0255 µM/mL) (Table 13). Notably, complex **C29** (0.0052 µM/mL) was the most effective agent compared to its counterparts, exhibiting a comparable activity to the reference drug (fluconazole) with a MIC value of 0.0051 µM/mL against *C. albicans*. The reactivity and inhibition trend against the tested microbes follows the sequence of **L^21−24^** < **C26** < **C27** < **C28** < **C29** ≤ fluconazole. The Cu(II) complexes exhibited the highest potency. Schiff base hydrazone ligands exhibited greater potency when coordinated with the Cu(II) ion [63].

The antifungal studies reviewed reveal that coordination of bioactive ligands with Cu(II) significantly enhances antifungal potency. This effect is commonly attributed to increased lipophilicity, improved cell permeability, and possible interaction with fungal biomolecules or the generation of reactive oxygen species.

Complexes such as **C19** and **C20** demonstrated superior activity against *Candida* and *Aspergillus* species, often outperforming standard drugs like ketoconazole and fluconazole. The enhanced efficacy is linked to ligand structure, coordination geometry, and nuclearity of the complexes. Schiff base-derived complexes (**C23**–**C29**) and those with pyrazole or morpholine-based ligands also exhibited more potent activity than their corresponding free ligands, supporting the chelation theory.

These results suggest that Cu(II) complexes are promising candidates for antifungal drug development. However, further studies, particularly the mechanisms of action, toxicity, and in vivo efficacy, are essential to advance them toward clinical applications.

## 4. Conclusions and Future Perspectives

In summary, this review highlights the promising antimicrobial potential of copper(II) complexes derived from various ligands, including Schiff bases, azoles, amino acids, and other nitrogen- and oxygen-donor systems. These complexes demonstrated significant antibacterial and antifungal activities, often outperforming or matching standard drugs in biological studies. The structure–activity relationships discussed provide insights into how ligand design, coordination geometry, and metal–ligand interactions influence biological efficacy.

Further research should focus on expanding the structural diversity of copper(II) complexes through synthesizing hybrid molecules, particularly those incorporating bioactive natural products or pharmaceutical scaffolds. Moreover, comprehensive in vivo studies and detailed toxicity assessments are essential to validate their therapeutic potential. Incorporating nanotechnology-based delivery systems may also enhance bioavailability and target specificity, opening new avenues in treating drug-resistant infections.

Overall, copper(II) complexes represent a versatile and underexplored class of antimicrobial agents with significant potential for future drug development. Continued interdisciplinary efforts in synthetic chemistry, microbiology, and pharmacology will be key to translating these findings into clinical applications.

## Figures and Tables

**Table 1:** Antibacterial activity of Cu(II) complexes (**C1**–**C3**) and their corresponding ligands (**L^1^**–**L^4^**).

Compounds	Antibacterial Activity		Antifungal Activity
	MIC (µg/mL)		
	*E. coli* (G^−^)	*B. cereus* (G^+^)	*A. fumigatus*
**L^1^**	75.50	77.50	67.50
**L^2^**	65.50	65.50	65.50
**L^3^**	54.50	53.50	56.50
**L^4^**	57.50	56.50	54.50
**C1**	21.34	22.50	20.50
**C2**	22.38	23.50	22.50
**C3**	22.35	23.50	21.50

**Table 2:** Antibacterial activity of complexes (**C4**–**C6**) and their corresponding ligands (**L^4^**, **L^6^**) (**C4**–**C6**) comparable to the ligands (**L^5^**–**L^7^**).

Compounds	MIC (MBC) (µg/mL)			
	Gram-Positive		Gram-Negative	
	*S. aureus* 29213	*S. epidermidis* 14990	*P. aeruginosa* 27853	*E. coli* 25922
**L^5^**	>256 (>256)	>256 (>256)	>256 (>256)	>256 (>256)
**L^6^**	>256 (>256)	>256 (>256)	>256 (>256)	>256 (>256)
**L^7^**	128 (>256)	256 (>256)	256 (>256)	128 (>256)
**C4**	128 (256)	128 (256)	128 (256)	128 (256)
**C5**	32 (32)	32 (32)	16 (16)	16 (32)
**C6**	16 (32)	16 (32)	8 (16)	8 (16)

**Table 3:** Antibacterial activity of complex (**C7**) and its corresponding ligand (**L^8^**).

Microbial Strains	Inhibition Zone (mm)/MIC (µg/mL)		
	L^8^	C7	Amoxicillin
*P. aeruginosa*	7/1000	19/62.5	28/12.5
*E. coli*	8/500	16/125	33/25
*S. sonnei*	9/500	21/62.5	26/125
*B. subtillis*	8/500	21/62.5	27/125

**Table 4:** Antibacterial activity of Cu(II) complexes (**C8**–**C10**) and their corresponding ligand 2-cetylpyridinenicotinichydrazone (**L^9^**).

		MIC (µg/mL)					
Compounds	*S. mutans*	*S. mitins*	*S. sanguinis*	*S. sobrinus*	*L. casei*	*S. salivarius*	*E. faecalis*
**L^9^**	1.56	>400	>400	400	400	3.12	>400
**C8**	6.25	400	>400	400	400	25	>400
**C9**	6.25	100	100	50	100	6.25	100
**C10**	3.12	100	200	50	100	6.25	100
Chlorhexidine	0.46	0.92	3.68	0.92	1.84	0.92	3.69

**Table 5:** Antibacterial activity of C(II) complexes **C11** and **C12**.

Compounds	Inhibition Zone (mm)			
	*S. aureus*	*S. pyogenes*	*E. coli*	*K. pneumonia*
**C11**	16 ± 0.2	15 ± 0.5	13 ± 0.6	13.5 ± 0.75
**C12**	13 ± 0.4	13.5 ± 0.3	12 ± 0.45	12.5 ± 0.6
DMSO	0 ± 0.00	0 ± 0.00	0 ± 0.00	0 ± 0.00
Gentamicin	25 ± 0.6	27 ± 0.75	26 ± 0.75	25 ± 0.5

**Table 6:** Antibacterial activity of complexes (**C13**–**C16**) and their corresponding ligands (**L^10^**, **L^13^**).

Compound	MIC (µg/mL)	
	*E. coli*	*S. aureus*
**L^10^**	512	256
**L^11^**	256	128
**L^12^**	128	64
**L^13^**	256	256
**C13**	128	32
**C14**	64	32
**C15**	64	32
**C16**	64	64
Streptomycin	8	4

**Table 7:** Antibacterial activity of complexes (**C17**, **C18**) and their corresponding ligands (**L^14^**, **L^15^**).

Compound	Inhibition Zone (mm)			
	Gram + ve		Gram − ve	
	*B. amyloliquefaciens*	*S. aureus*	*E. coli*	*K. pneumoniae*
**L^14^**	01± 0.2	02 ± 0.3	0	01 ± 0.1
**L^15^**	0	01 ± 0.4	0	01 ± 0.4
**C17**	26 ± 0.2	23 ± 0.4	21 ± 0.3	22 ± 0.2
**C18**	25 ± 0.2	23 ± 0.1	20 ± 0.2	21 ± 0.3
Ampicillin	30 ± 0.2	31 ± 0.2	30 ± 0.2	30 ± 0.2

**Table 8:** Antifungal activity of Cu(II) complex **C19** in comparison with ketoconazole.

Compound	MIC (µg/mL)		
	*C. albicans* ATCC 14053	*C. krusei* ATCC 34135	*C. glabrata* ATCC 2001
**C19**	130	66	260
ketoconazole	470	7.3	235

**Table 9:** Antifungal activity of Cu(II) **C20**–**C22** and their corresponding ligand **L^17^**.

MIC (µg/mL)								
Compounds	Strains							
	*C. albicans*	*C. krusei*	*C. glabrata*	*C. neoformans*	*A. niger*	*A. Terreus*	*A. fumigatus*	*A. flavus*
**C20**	0.2	0.2	0.1	0.05	0.05	0.2	0.1	0.05
**C21**	0.4	0.4	0.1	0.1	0.1	0.4	0.1	0.1
**C22**	0.2	0.4	0.2	0.1	0.1	0.2	0.2	0.2
**L^17^**	0.4	0.4	0.2	0.2	0.2	0.4	0.2	0.2

**Table 10:** Antifungal activity of complexes (**C23**, **C24**) and their corresponding ligands (**L^18^**, **L^19^**).

Compound	Inhibition Zone (mm)	
	*Sclerotium rolfsii*	*Macrophomina phaseolina*
**L^18^**	8	10
**L^19^**	9	7
**C23**	23	22
**C24**	22	20
Mancozeb	30	31

**Table 11:** Antifungal activity of Cu(II) complex **C7** and its corresponding pyrazole-based nucleating ligand (L^8^) in comparison with fluconazole.

Microbial Strains	Inhibition Zone (mm)/MIC (µg/mL)		
	L^8^	C7	Fluconazole
*A. niger*	13/500	23/125	28/62.5
*A. flavus*	12/500	25/125	21/125
*F. oxysporum*	10/500	17/125	20/125
*P. notatum*	8/500	19/125	20/125

**Table 12:** Antifungal activity of Cu(II) complex **C25** and its corresponding bidentate-morphine-based ligand (**L^20^**) in comparison with amphotericin.

Compounds	Inhibition Zone (mm)	
	*C. albicans*	*A. niger*
**L^20^**	13.3 ± 0.67	11.7 ± 0.59
**C25**	23.7 ± 1.19	19.8 ± 0.99
amphotericin	28.7 ± 1.44	28.6 ± 1.43

**Table 13:** Antifungal activity of Cu(II) coordinated complexes (**C26**–**C29**) and their Schiff-base ligands (**L^21^**–**L^24^**).

Compounds	MIC(µg/mL)	
	*C. albicans*	*A. niger*
**L^21^**	0.0449	0.0449
**L^22^**	0.0399	0.0399
**L^23^**	0.0328	0.0328
**L^24^**	0.0301	0.0301
**C26**	0.0068	0.0137
**C27**	0.0063	0.0255
**C28**	0.0056	0.0112
**C29**	0.0052	0.0105
Fluconazole	0.0051	0.0102
